# Seasonal Migratory Activity of the Beet Armyworm *Spodoptera exigua* (Hübner) in the Tropical Area of China

**DOI:** 10.3390/insects15120986

**Published:** 2024-12-12

**Authors:** Xudong Wang, Qing Feng, Xianyong Zhou, Haowen Zhang, Shaoying Wu, Kongming Wu

**Affiliations:** 1 School of Tropical Agriculture and Forestry, Hainan University, Haikou 570228, China; 15565306888@163.com (X.W.);; 2 The State Key Laboratory for Biology of Plant Diseases and Insect Pests, Institute of Plant Protection, Chinese Academy of Agricultural Sciences, Beijing 100193, China; 3 College of Plant Protection, Fujian Agriculture and Forestry University, Fuzhou 350002, China

**Keywords:** *Spodoptera exigua*, migration trajectory, ovary development, regional management

## Abstract

The beet armyworm *Spodoptera exigua* (Hübner), a global pest, feeds on and affects a wide range of crops. Its long-distance migration with the East Asian monsoon frequently causes large-scale outbreaks in East and Southeast Asia. This pest mainly breeds in tropical regions in the winter season every year; however, few studies have investigated associations with its population movements in this region. From 2017 to 2023, we monitored its population dynamics in a tropical site, located in Hainan Province of China, using a searchlight trap. Dissection of the ovaries of female *S. exigua* moths captured from the air revealed that most of them were reproductively mature and could be classified as a transit migratory population. Migration occurred most often in summer and least often in winter, with an increasing trend over the years. According to a trajectory model analysis based on the Weather Research and Forecasting (WRF) model, *S. exigua* migrated from Hainan Island to mainland China in the spring, primarily moved from the areas of Southeast Asia to Hainan and mainland China during the summer, and returned from China to Southeast Asia in the autumn and winter. Overall, our research defines the movement paths of *S. exigua* in the tropical area of China, establishing a theoretical foundation for its regional monitoring, early warning, and management in China and Southeast Asian countries.

## Simple Summary

*Spodoptera exigua* is a migratory pest that causes regional crop loss regularly. In this study, its migratory activity was monitored using a searchlight trap in the tropical area of Hainan province, China. The results indicated that the pest migrated from Hainan Island to mainland China in the spring, primarily moved from areas of Southeast Asia to Hainan and mainland China during the summer, and returned to Southeast Asia from China in the autumn and winter. These findings provide a theoretical basis for the regional monitoring, early warning, and management of the insect in China and Southeast Asian countries.

## 1. Introduction

*Spodoptera exigua* (Hübner) (Lepidoptera: Noctuidae) is a widespread agricultural pest originating in South Asia [1]. It has an annual distribution range of approximately 101 countries and regions worldwide [2,3], with approximately 70% of these located in Asia and Africa [4]. Its larvae feed on the leaves and fruits of 170 host plants [5], including vegetables, cotton, corn, beans, tobacco, alfalfa, flowers, and fruit trees [6,7]. *S. exigua* frequently migrates over great distances [8,9] with the longest documented continuous flight being 3500 km [10]. It also has a high reproductive capacity [11]. These biological traits greatly contribute to outbreaks of *S. exigua*. The migration of *S. exigua* from Southeast Asian countries to China has been tracked over the South China Sea, while research in China’s Bohai Bay area reveals seasonal cross-sea migration patterns [12,13]. Recent studies have shown that the genetics and geographical distance of *S. exigua* populations are not correlated, with no significant differences found between populations, indicating that *S. exigua* populations in China have expanded recently [14,15,16].

*S. exigua* has become increasingly destructive over the last two decades [17,18,19]. In China, it has severely harmed several farmed crops, including cabbage, cowpeas, soybeans, onions, and cotton [20,21,22], causing significant losses in agricultural output [23,24]. Since the 1990s, extensive research has been performed in China on its migration and overwintering patterns, as well as its behavioral mechanisms. The results have shown that *S. exigua* survives in winter through two key strategies: migration and overwintering [13,18]. As a result, monitoring and early warning for its migration activities is critical for developing prevention and control strategies. Applying meteorological concepts to examine insect migration trajectories is also an essential method for studying the origins of migratory insect populations and predicting migration areas [25,26,27]. The Weather Research and Forecasting (WRF) model is a next-generation meteorological data simulation model that is widely used to simulate migration paths [28]. The WRF model’s trajectory simulation, when combined with the insect’s biological parameters, is highly accurate, and plays an essential role in the early detection and control of several key agricultural pests, such as *Spodoptera frugiperda* [29,30].

Studies have shown that *S. exigua* cannot overwinter in China’s Jianghuai and Yangtze River basins [31,32]; instead, they overwinter in locations south of the 12 °C isotherm, such as the Guangdong, Fujian, and Hainan provinces, inflicting damage all year round. In certain regions, the pest not only damages crops and breeds all year, but it also moves to inflict damage further inland. However, few studies have investigated *S. exigua* ‘s association with population movements in China’s tropical region. In this study, we used high-altitude searchlights to monitor the population dynamics of *S. exigua* in Lingshui County, Hainan Province, from 2017 to 2023. We dissected the ovaries of captured female *S. exigua* moths, and simulated their trajectories. Our findings clarify the seasonal migration patterns of *S. exigua* in southern China and Southeast Asia, establishing a theoretical foundation for its regional monitoring, early warning, and management in China and Southeast Asian countries.

## 2. Materials and Methods

### 2.1. Population Monitoring Site

Monitoring stations are installed at the Modern Agriculture Demonstration Base (18°26′ N, 109°50′ E) in Lingshui County, Hainan, an important southern breeding research site in China. This site is used to develop crop varieties suitable for the climate, soil, and ecological environment of the southern region, thereby increasing agricultural production [33]. This region has a typical tropical monsoon ocean climate, with extended daylight hours, adequate sunshine, and modest temperature variations between day and night. High temperatures above 30 °C are the most typical from April to September. Plenty of tropical crops and off-season vegetables grow in this region.

### 2.2. Searchlight Moth Trapping Method

**Figure 1:**
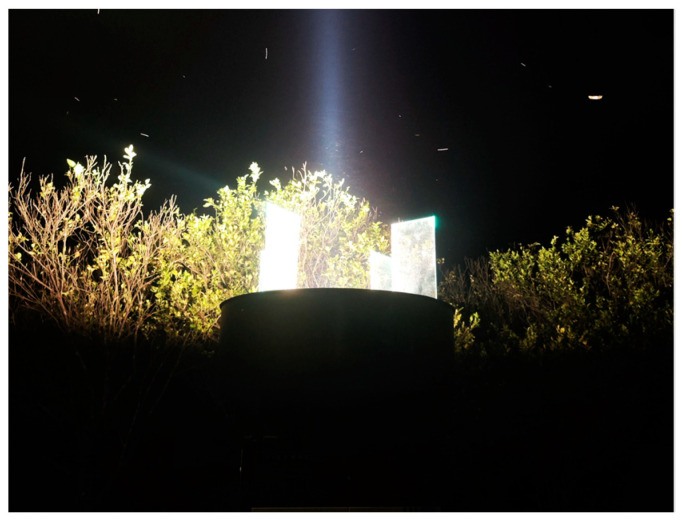
Working status of the searchlight traps.

Vertically oriented high-altitude searchlight traps were used to attract and collect insects. These searchlights can capture phototactic insects up to a height of 500 m, and have been found to be beneficial for monitoring migratory pest population dynamics [34]. The apparatus included a GT75 searchlight with a JLZ 1000 W metal halide bulb (Shanghai Yaming Lighting Co., Ltd., Shanghai, China; flux 90,000 Lm, color temperature 4000 K, and color-rendering index 65), and a 60-mesh nylon net suspended beneath the trap. Bags were used to collect the captured insects (Figure 1). The monitoring period was from 19 December 2016 to 31 September 2023. Besides the equipment maintenance period in the second half of 2019, the light traps continued to operate throughout the period after 2017 (except in conditions of extreme weather or power shortage). Every day, the lights were turned on at sunset and turned off at sunrise the following day. The insect trap net bags collected each day were placed in a −20 °C freezer. The captured insect samples were classified based on their morphology, from which the *S. exigua* was identified, counted, and preserved.

### 2.3. Ovarian Dissection

Insect migration is directly linked to ovarian development. Migrating populations show higher female development than that in sedentary local populations [35]. This study used the method described by Wang et al. [36] to grade the ovarian development of *S. exigua*. The ovaries of *S. exigua* females were divided into four developmental stages: yolk deposition (level one), mature spawning (level two), peak egg laying (level three), and termination of egg laying (level four). The ovaries were examined for mating. The *S. exigua* females were considered to have mated as many times as the number of spermatophores in the mating sac. The absence of mating was regarded as an unmated mating sac; otherwise, one or more mating episodes were documented. The developmental stage was documented as well as number of mating episodes [37]. The observations were performed using a trinocular stereoanatomical microscope (SWG-L45B from Shenzhen Sweiguan Optical Instrument Co., Ltd., Shenzhen, China).

### 2.4. Trajectory Simulation

Trajectory analysis primarily employed the Weather Research and Forecasting (WRF) model, a new-generation meteorological data simulation model developed collaboratively by several research institutions, including the National Centers for Environmental Prediction (NCEP) and the National Center for Atmospheric Research (NCAR). In this study, InsectTrace, a trajectory simulation model based on the WRF meteorological data, was used for simulation (Table 1) [25]. This model was designed based on the FORTRAN language [38].

Final Analysis (FNL) data were derived from the NCEP and NCAR, and were used to initiate the initial field data and boundary conditions of the WRF model. A 10 km × 10 km grid-spaced meteorological element field was output once every hour as the background condition to drive the calculation of insect migration paths, after the meteorological data were fed into the WRF model and simulated.

Based on the biological characteristics of *S. exigua*, the following migration traits and parameters were defined in the trajectory analysis: (1) the trajectory simulation end time was 06:00 on the next day, and the start time was 20:00 Beijing time [39]; (2) they flew with the wind at high altitude, regardless of their own flight speed and directional deflection angle [40]; (3) according to the terrain characteristics, the flight altitude was set as 100–1000 m above the ground altitude, a range that covers the typical altitudes of migratory insects [41], and every 100 m was an interval, resulting in a total of 10 intervals; (5) as soon as the upper air temperature fell below 10 °C, the simulation ended [42,43]. The migration trajectories were visualized using ArcGIS10.7 (Esri, Redlands, CA, USA). A map was generated by inputting the coordinate data points obtained by WRF.

### 2.5. Data Analysis

All the data were normalized using the Shapiro–Wilk test and Levene’s test for variance homogeneity. Percentage data were evaluated using the square root arcsine transformation. A general linear model was used to investigate the mating rate, ovarian development level, number of mating episodes, and trapping numbers of the *S. exigua* females [44]. When a significant difference was found, Tukey’s HSD was applied. Each month, the sex ratio was compared to the theoretical sex ratio (1:1) using a chi-square test. Analyses were performed using SPSS 26.0 (IBM, Armonk, NY, USA). Fisher’s optimal clustering method [45] was used to conduct an optimal time pattern analysis of seasonal fluctuations in the population, for the average weekly total trapped numbers of *S. exigua* from 2017 to 2023, using the statistical software Data Processing System 13.0 [46].

## 3. Results

### 3.1. Seasonal Migration Dynamics of S. exigua

**Figure 2:**
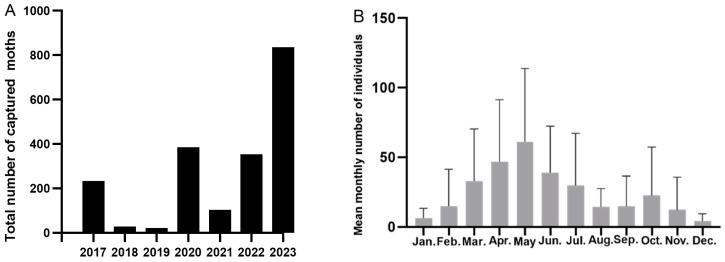
Total annual catches (**A**) and mean monthly catches (**B**) of *S. exigua* during 2017–2023.

From 2017 to 2023, 1961 adults were captured at the Lingshui site. *S. exigua* could be captured every month of the year; however, trapping amounts varied significantly by year and month (year: F = 5.715, *p* < 0.001; month: F = 3.067, *p* = 0.003). The maximum trapping volume of *S. exigua* was recorded in 2023 (836 adults), followed by 2020 (385 adults) and 2022 (353 adults), with the lowest in 2019 (22 adults) (Figure 2A). Over the last seven years, the monthly trapping volume of *S. exigua* was the highest in May (61.14 adults), followed by April (39.71 adults), and it was the lowest in January and December (<10 adults) (Figure 2B).

**Figure 3:**
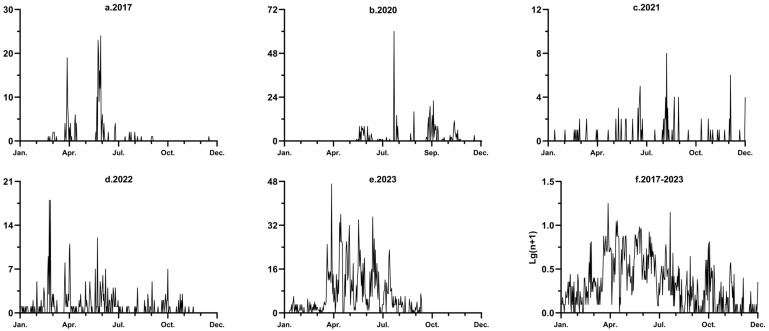
Nightly catches from 2017 (**a**) and 2020 to 2023 (**b**–**e**), and mean logarithm numbers (**f**) of *S. exigua* moths captured in light traps.

The monthly migration pattern was divided into five periods (Table 2): the spring migration season (late March to mid-May); the summer migration season (late May to early June); the autumn–winter migration season (August to December); migration transition period I (January to early March), which had a lower trapping volume; and migration transition period II (June–July), which had a higher trapping volume, because the error function value decreased at the maximum rate when k = 5. Although the trapping volume fluctuated substantially from year to year, the annual daily population dynamics (years 2018–2019 were excluded, since the average monthly catches were less than 5) suggest that *S. exigua* continued to migrate seasonally (Figure 3).

### 3.2. Sex Ratio and Status of Ovarian Development in Aerial Trapping of S. exigua

**Figure 4:**
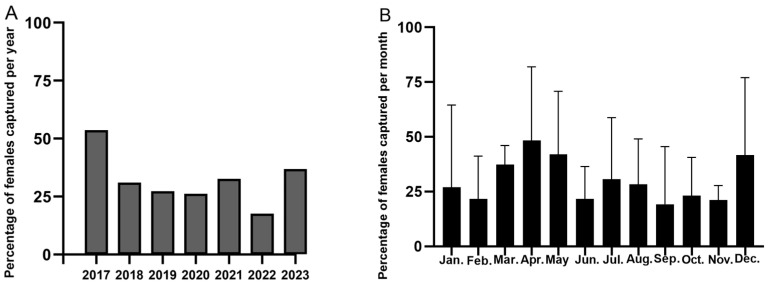
Percentage of annual (**A**) and monthly (**B**) trapped *S. exigua* females.

From 2017 to 2023, the female trapping rate of *S. exigua* increased before declining (Figure 4). April had the highest monthly average female proportion (48.30%). The proportion of females was more stable in July and August, at approximately 30%. Except for April–May and December, the average female rate in the other months was less than 40%. A chi-square test revealed that considerably more males were trapped than females from January to November, but not in December (Table 3). Analysis of variance revealed a significant difference in the female proportions of *S. exigua* in different years (year: F = 3.599, *p* = 0.004), but no significant difference in months (month: F = 1.525, *p* = 0.153).

**Figure 5:**
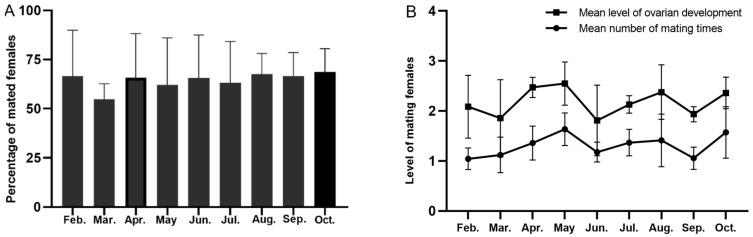
Ovarian development status of trapped *S. exigua* females monthly from 2021 to 2023. (A) Percentage of mated females. (**B**) Mean level of ovarian development and number of mating times.

**Figure 6:**
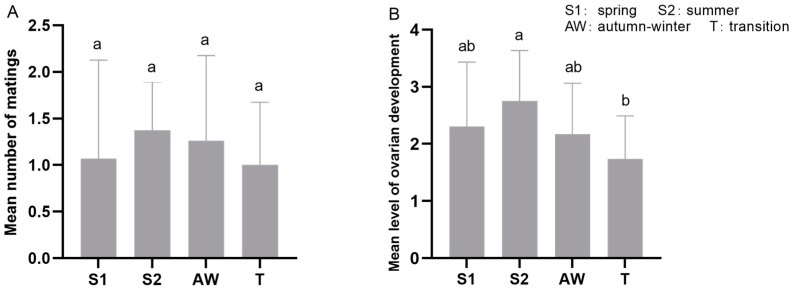
The number of matings (**A**) and level of ovarian development (**B**) of *S. exigua* females during migration periods. Different lowercase letters above clustered columns indicate significant differences (*p* < 0.05).

A total of 220 female insects from 2021 to 2023 were dissected. Overall, the average ovary development grade, mating rate, and number of matings in some months between 2021 and 2023 were higher (Figure 5). Among the four migration periods, the ovary development level of female moths in the summer migration period was significantly higher than that in transition period (F = 2.580, *df* = 3, *p* = 0.056),while there was no significant difference in mating frequency (F = 0.544, *df* = 3, *p* = 0.653) (Figure 6).

Moreover, the average mating rate of the females in each month exceeded 60%, except for in March, with the rate in October being the highest, at 68.5% (Figure 5A). Analysis of variance revealed that the year and month had a significant effect on the mating rate (year: F = 5.831, *p* = 0.013; month: F = 16.947, *p* < 0.001).

The ovarian development grade and the number of matings per month showed a positive correlation: the higher the ovarian development grade, the more likely mating was to occur. Besides March, June, and September, females had an average ovarian development grade higher than level 2, and more than one mating event. As Figure 5B shows, females had the highest ovarian development grade (~2.5) and mating times (~1.7 times) in April–May. The year had a substantial effect on ovarian development grade and the number of matings (F = 4.046, *p* = 0.033; F = 3.833, *p* = 0.037), as did the month (F = 3.691, *p* = 0.005; F = 3.434, *p* = 0.007).

### 3.3. Trajectory Simulation of Population Migration Paths

**Figure 7:**
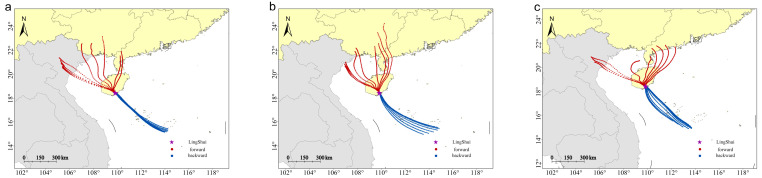
Simulated migration trajectories of *S. exigua* moths, on peak days in the spring season. (**a**–**c**): 2021, 2022, and 2023, respectively.

**Figure 8:**
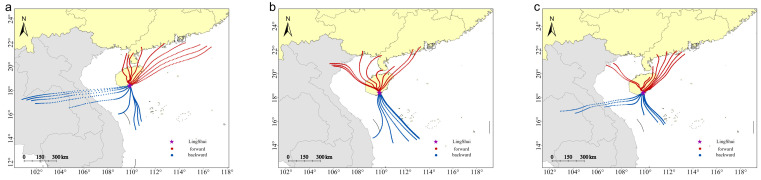
Simulated migration trajectories of *S. exigua* moths on peak days in the summer season. (**a**–**c**): 2020, 2022, and 2023, respectively.

**Figure 9:**
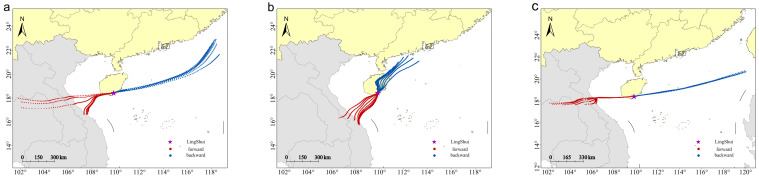
Simulated migration trajectories of *S. exigua* moths on peak days in the autumn–winter season. (**a**–**c**): 2020, 2021, 2022, respectively.

The forward and backward simulation trajectories for 12 h on the peak day of the annual migration reveal distinct flight patterns for *S. exigua* during different migration periods. These include the peak migration period (late May to early June), the spring migration period (late March to mid-May), and the autumn and winter migration period (August to December). The trajectories show migration within Hainan Island and periodic round-trip migration between China and Vietnam. During the spring migration period, *S. exigua* was primarily a local population on the island, because the backward trajectories did not reach any source areas. Forward trajectories pointed northwards in the interior of China, suggesting northward travel to the interior of China (Figure 7). During the peak summer migration season, the main migration routes were Indochina–Hainan and China–Mainland China, with an overall migration direction from west to east, and a maximum migration distance of approximately 550 km (Figure 8). The migratory route ran in the opposite direction during the autumn–winter migration period compared with that in the peak summer migration period (Figure 9), causing the population from Hainan to spread to Vietnam and Laos.

## 4. Discussion

Previous research has demonstrated that *S. exigua* migrates seasonally in some locations in Bohai Bay in northern China [13], but its cross-border migration behavior between southern Hainan and Indochina has remained unknown. In this study, we confirmed that *S. exigua* engages in long-distance seasonal migration between Indochina and China, with a reciprocating movement pattern from southwest to northeast. Long-term continuous monitoring of population dynamics in Lingshui Base, Hainan Province, from 2017 to 2023 revealed significant seasonal changes in the *S. exigua* population, which can mainly be divided into three migration periods: spring, summer, and autumn–winter. The ovarian dissection of female *S. exigua* trapped using high-altitude lights revealed that the ovary development level during the spring migration period was lower, possibly because of the decrease in the local population, whereas the ovary development level during the summer migration period, as well as the autumn and winter migration periods, was higher, indicating that most of the trapped *S. exigua* migrated into Hainan. Furthermore, the male-to-female ratio revealed that the number of females was much lower throughout the year than the number of males. Finally, we confirmed that *S. exigua* engages in long-distance seasonal migration between Indochina and China, with a reciprocating movement pattern from southwest to northeast. The WRF model trajectory analysis revealed the cross-border migration pattern of *S. exigua* between southern China and Southeast Asian countries.

Flying does not reduce the mating ability of the *S. exigua*, a phenomenon also seen in other species such as the *Autographa nigrisigna* [47,48,49]. The ovarian development status of female insects caught by high-altitude lights can be used to determine the kind of migration [28]. In the present study, during the migration period of *S. exigua*, the number of captured males was much higher than that of females, similar to findings from the migration of *S. exigua* over Bohai Bay in China [13]. In this study, the population that migrated from Vietnam to Hainan during the peak summer migration period (late May to early June) had a higher level of ovarian development, and the number captured was the highest of the year, indicating that it is a typical migratory population. *S. exigua*’s ovaries mature at the peak of migration, and the adults’ migration and reproduction can occur simultaneously and do not inhibit each other, contradicting the “oogenesis and flight conjugation” theory. However, during migration, females may finish their trip early owing to oviposition activities, resulting in poor trapping rates [35,50,51,52]. The migratory behavior of *S. exigua* does not interfere with its reproductive ability, as the ovaries could develop during migration, which provides a physiological basis for rapid reproduction after arriving in the breeding area [53]. Corn, beans, and other crops grown in southern Hainan throughout the autumn and winter provide adequate food for *S. exigua* reproduction, but these crops are usually planted in a single season. After harvest, the loss of habitat prompts the local population to migrate.

Hainan Island serves as a transit point for insect migration between China and other Southeast Asian countries [54]. Some important agricultural migratory pests, such as *S. litura* [55], *S. frugiperda* [42], and rice planthoppers [56], also show evident migration peaks in Hainan; however, their peak seasons differ. Maize, the major host crop of *S. exigua*, is planted at different times throughout its migration path between China and Southeast Asia, depending on the regional climate and soil conditions [52]. The migratory biomass of *S. exigua* varies significantly between years, and it has increased rapidly in recent years, with the majority of the population migrating in the spring and summer. Maize is planted annually in northern Vietnam from April to May, and the maize-growing area in Vietnam is increasing [57], creating favorable conditions for *S. exigua* cross-border migration, as it is a good food source for *S. exigua* larvae. Additionally, trajectory research reveals that the prevailing southwesterly wind helps *S. exigua*’s cross-border migration to Hainan. *S. exigua* can reproduce year-round in East Asia, including in the tropical climate zone (TCZ) and the southern part of subtropical climate zone (STCZ) occurrence regions (<25° N) [14,32]. Corn is mainly planted throughout the winter and spring in Hainan. Corn planting and favorable climatic conditions in Hainan make it an ideal overwintering location for migrating *S. exigua*. These findings are consistent with those of Zhou’s [12] regarding insect pest migration from Southeast Asian countries to China via Hainan.

The East Asian monsoon is an essential component of the global climate system that regulates seasonal changes in wind, rainfall, terrestrial vegetation, and other climatic and environmental variables throughout East Asia [58,59]. East Asian monsoon airflow encourages many insects to migrate long distances across borders, this study provides further documentation on pest movement along the extensive Southeast and East Asian Insect Migration “Flyway” [60,61], and heavy monsoon activity supports the seasonal movement of *S. exigua* [62]. According to the “Climate Change 2022: Impacts, Adaptation, and Vulnerability” report by the IPCC [63], Asia is one of the most vulnerable regions regarding the effects of climate change, and pest migration outbreaks are likely to continue with the integrated development of global climate change and agricultural production. The FAO launched the “Global Action for the Prevention and Control of Fall Armyworms” guide in December 2019, which can guide global efforts to prevent and control major crop pests, including *S. exigua*.

However, due to its migration behaviors and the rapid development of pesticide resistance, controlling *S. exigua* is becoming more challenging [64]. Based on migration patterns and monitoring results, nocturnal insect phototaxis can be used to influence insect flight through light intensity [65], along with trapping and eliminating aerial migratory populations using high-altitude lights [66]. This approach is commonly utilized for early warning of the long-distance migration activities of migratory pests [15,67]. Establishing a comprehensive monitoring, early warning, prevention, and control system is an effective way of mitigating the harm caused by migrating pests. Early monitoring, early warning, and prompt prevention and control can successfully reduce the insect population, avoid the use of chemical pesticides, and lower the risk of pesticide resistance in *S. exigua* [68]. This study aimed to investigate the migration and spread of *S. exigua* in China, using cross-regional migration across Southeast Asian nations as an example. These findings can help better understand the patterns of *S. exigua* migration in different climate zones, as well as improving pest monitoring and early warning capabilities for its cross-border migration. In future studies, we will integrate other methodologies, such as stable isotope tracing, genetic diversity detection, and Entomological Radar Monitoring, to better understand the migration process of *S. exigua*. Continued monitoring and collaboration with Southeast Asian countries is necessary to predict the origins and migration routes of insects, and field investigations are needed to validate the predicted results.

## 5. Conclusions

This study revealed that *S. exigua* migrates seasonally across southern China and Southeast Asia. Therefore, establishing a sustainable joint prevention and control system for East and Southeast Asia is critical. Furthermore, international collaboration should be increased through monitoring and early warning networks to improve joint prevention and control among countries and regions and to better handle worldwide pest and disease challenges.

## Figures and Tables

**Table 1:** Scheme and parameters of the WRF model.

Item	Domain
Number of grid points	200 × 200
Distance between grid points	10 km
Layers	32.00
Map projection	Lambert
Microphysics scheme	Thompson
Longwave radiation scheme	RRTMG
Shortwave radiation scheme	RRTMG
Surface layer scheme	Monin–Obukhov
Land/water surface scheme	Noah
Planetary boundary layer scheme	Mellor–Yamada–Janjic
Cumulus parameterization	Tiedtke

**Table 2:** Optimal segmentation of groups based on the weekly number of *S. exigua* captured in light traps during 2017–2023.

Divide Number	Optimal Segmentation	Error Function	(*df*_1_, *df*_2_)	Pseudo_F	Slope_Ratio
2	1–30, 31–52	38.8	1, 50	7.2	1.0
3	1–11, 12–25, 26–52	23.9	2, 49	18.5	3.8
4	1–11, 12–20, 21–22,23–52	19.9	3, 48	27.7	0.6
5	1–11, 12–20, 21–22, 23–29, 30–52	12.2	4, 47	19.3	3.9
6	1–11, 12–20, 21–22, 23–29, 30–41, 42–52	9.7	5, 46	27.9	0.6

**Table 3:** Percentage of females captured per month.

Month	No. (%)	*x* ^2^	*p*	Month	No. (%)	*x* ^2^	*p*
Jan.	9(20.5)	8.417	0.004	Jul.	61(30.0)	16.868	<0.001
Feb.	33(31.1)	7.826	0.005	Aug.	33(32.0)	6.898	0.009
Mar.	98(41.0)	3.980	0.048	Sep.	38(34.9)	5.135	0.023
Apr.	101(36.5)	10.361	0.001	Oct.	23(22.3)	17.148	<0.001
May	161(37.1)	14.696	<0.001	Nov.	10(14.9)	18.898	<0.001
Jun.	76(29.7)	22.034	<0.001	Dec.	8(40.0)	0.404	0.525

## Data Availability

Data will be made available on request.
